# Refractive index variation in a free-standing diamond thin film induced by irradiation with fully transmitted high-energy protons

**DOI:** 10.1038/s41598-017-00343-0

**Published:** 2017-03-24

**Authors:** S. Lagomarsino, S. Calusi, M. Massi, N. Gelli, S. Sciortino, F. Taccetti, L. Giuntini, A. Sordini, M. Vannoni, F. Bosia, D. Gatto Monticone, P. Olivero, B. A. Fairchild, P. Kashyap, A. D. C. Alves, M. A. Strack, S. Prawer, A. D. Greentree

**Affiliations:** 1 Department of Physics and Astronomy, University of Firenze, Firenze, Italy; 2 Istituto Nazionale di Fisica Nucleare (INFN), Sezione di Firenze, Firenze, Italy; 3 Department of Experimental and Clinical Biomedical Sciences “Mario Serio”, University of Firenze, Firenze, Italy; 4 Istituto Nazionale di Ottica (INO), CNR, Firenze, Italy; 5 Physics Department and NIS Inter-departmental Centre, University of Torino, Torino, Italy; 6 Istituto Nazionale di Fisica Nucleare (INFN), Sezione di Torino, Torino Italy; 7 Consorzio Nazionale Interuniversitario per le Scienze fisiche della Materia (CNISM), Sezione di Torino, Torino Italy; 8 School of Physics, University of Melbourne, Melbourne, Australia; 9 Australian Research Council Centre of Excellence for Nanoscale BioPhotonics, RMIT University, Melbourne, 3001 Australia; 10 Royal Melbourne Institute of Technology (RMIT), Melbourne, Australia; 11 European XFEL GmbH, Hamburg, Germany

## Abstract

Ion irradiation is a widely employed tool to fabricate diamond micro- and nano-structures for applications in integrated photonics and quantum optics. In this context, it is essential to accurately assess the effect of ion-induced damage on the variation of the refractive index of the material, both to control the side effects in the fabrication process and possibly finely tune such variations. Several partially contradictory accounts have been provided on the effect of the ion irradiation on the refractive index of single crystal diamond. These discrepancies may be attributable to the fact that in all cases the ions are implanted in the bulk of the material, thus inducing a series of concurrent effects (volume expansion, stress, doping, etc.). Here we report the systematic characterization of the refractive index variations occurring in a 38 µm thin artificial diamond sample upon irradiation with high-energy (3 MeV and 5 MeV) protons. In this configuration the ions are fully transmitted through the sample, while inducing an almost uniform damage profile with depth. Therefore, our findings conclusively identify and accurately quantify the change in the material polarizability as a function of ion beam damage as the primary cause for the modification of its refractive index.

## Introduction

Diamond is emerging as a promising platform for the development of integrated photonic devices, due to the appealing properties (quantum efficiency and photo-stability, spin-preserving transitions and room temperature operation) of a vast range of defect-related colour centres^1–5^, which can be incorporated in the broadly transparent crystal matrix of diamond by ion implantation^6–8^. Different integrated photonic devices were created in diamond to take advantage of the unique properties of the above-mentioned centres by means of different microfabrication strategies, many of which rely on the use of energetic ion beams to both fabricate photonic microstructures^9–13^ and fine-tune their refractive index^14, 15^. The structural effects of both MeV ion beam^15^ and femto-second laser pulse^16, 17^ irradiations have been used to directly write waveguides in bulk diamond. In particular, Focused Ion Beam (FIB) techniques are well-established in the fabrication of optical/photonic nanostructures in diamond^18, 19^. In this context, the accurate control over both intentional and unintentional variations of the refractive index of the material is of paramount importance for the development of photonic devices with the desired functional properties.

The effect of ion-induced structural damage on the refractive index in diamond was observed for the first time in the 60 s^20^ and qualitative observations have more recently been reported^21^. However, only in recent years has this process been investigated more systematically with different characterization techniques and various ion energies and species.

In the case of deep-penetrating (i.e. ~1–50 µm) MeV light ions, laser interferometric microscopy was employed to study the effects of 2–3 MeV H^+^ implantation at increasing fluences^22, 23^, while Coherent Acoustic Phonon spectroscopy (CAP) was used to characterise samples implanted at increasing fluences with 1 MeV He^+^^24, 25^. In the case of less penetrating (i.e. 15–350 nm) 10–100 keV ions, spectroscopic ellipsometry was employed to characterise variations in refractive index in samples implanted respectively with 350 keV He^+^^26^, 180 keV B^+^^27^ and 30 keV Ga^+^^28, 29^ ions.

In all of the above-cited works, the ion energy employed for the irradiation was such that the irradiated ions came to rest in a thick target at different depths, depending upon the ion species and energies. This fact introduces a level of complication in the interpretation of the experimental results, due to the strongly non-uniform depth profile of the ion induced damage. This effect is more pronounced for MeV ions and it can seriously affect the interpretation of measurements performed with interferometric microscopy and CAP. This is due to the large gradients in refractive index occurring near the end-of-range depth of the ions, determining complex optical effects on the probe laser beam that are not easy to account for. Although less pronounced for keV ion implantations, the inhomogeneity in the depth distribution of induced damage requires the implementation of multi-layer modelling in the analysis of ellipsometric data^30^.

The issue of a non-uniform damage density depth profile^31^ was addressed in ref. 32, where carbon ions were implanted at different energies (0.05–1.5 MeV) and fluences (1.5 × 10^14^–7.5 × 10^16^ cm^−2^) with the purpose of generating a uniformly damaged layer. Although beneficial, this strategy is potentially limited by the fact that multiple-energy ion implantations are more time-consuming, and that it is very complicated to duly take into account the non-linear effects related to the mutual interactions of nuclear and electronic energy losses from different implantations^33^.

Another important consideration is that the full stopping of implanted ions in the target material can potentially introduce doping effects when foreign ion species are implanted into the diamond matrix. The doping constitutes a further experimental variable that could play a role in determining the significant discrepancies observed in the results reported in the above-cited works. For example, contradictory results were found even on the qualitative trend of the refractive index variation as a function of the damage density: in some works a monotonic increase was reported^20, 22, 23, 27^, while other works reported a decreasing variation^25^ or a non-monotonic behaviour^28, 29, 32^.

In the present work, we report on the systematic characterization of the damage-induced refractive index variations at *λ* = 632.8 nm in a µm-thin diamond sample through which the irradiated ions (i.e. protons at 3 MeV and 5 MeV) are fully (i.e. >99.9%) transmitted. This approach significantly simplifies the interpretation of the experimental data, for several reasons. Firstly, it allows the definition of an extremely uniform damage profile across the sample thickness. Secondly, all the issues associated with the ion end of range (namely: doping-related effects from the implanted ions, abrupt variations in damage density, complex effects due to the interplay between electronic and nuclear energy loss^33^) are significantly minimised.

## Results

**Figure 1:**
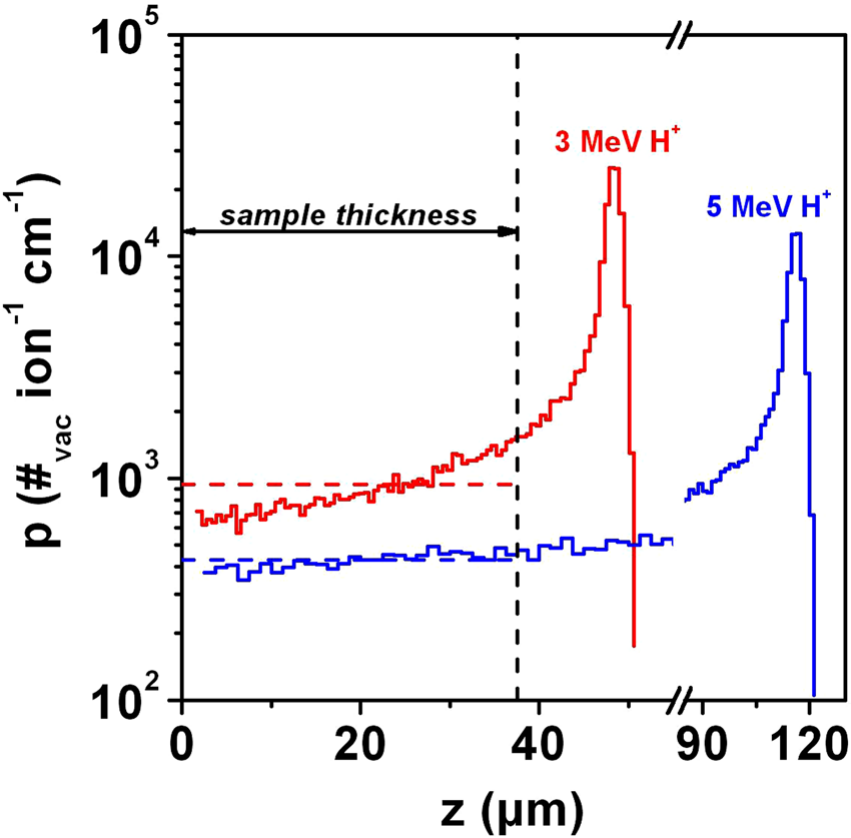
Ion damage profile. Depth profiles of linear vacancy concentration per single ion and unit length across the diamond sample thickness, as evaluated with SRIM SRIM-2008.04 Monte Carlo code for both 3 MeV (red plot) and 5 MeV (blue plot) proton irradiation. The vertical dashed line marks the thickness of the diamond sample, while the relevant average values across the sample thickness are reported by the horizontal dashed lines. Maximum and minimum values of the linear vacancy concentration across the sample thickness are: ~1.5 × 10^3^ cm^−1^ and ~7 × 10^2^ cm^−1^ for 3 MeV ions, ~4.7 × 10^2^ cm^−1^ and ~3.8 × 10^2^ cm^−1^ for 5 MeV ions.

The sample was irradiated with protons having energies of 3 MeV and 5 MeV. The depth range of 3 MeV and 5 MeV protons in diamond is respectively ~50 µm and ~115 µm, to be compared to the 38 µm thickness of the sample (see Fig. 1). Therefore, as evaluated with SRIM-2008.04 Monte Carlo code^34^, in both cases >99.9% of the ions are fully transmitted through the sample thickness. In Fig. 1, the SRIM-determined depth profiles of the linear vacancy concentration *p*(*z*) are reported for both 3 MeV and 5 MeV protons. The linear concentration values *p*(*z*) remain fairly constant within the thickness of the diamond samples, while avoiding the damage peak occurring at the ions’ end of range. Fluence values ranged between ~1 × 10^15^ cm^−2^ and ~1 × 10^18^ cm^−2^. Its experimental uncertainty is estimated to be ~5%, on the basis of the uncertainties on the measured ion current and on the size of the irradiated areas. Amorphisation is known to occur in diamond when the strain introduced by ion-implantation-induced damage overcomes a critical threshold, corresponding to the building up of critical stresses in the material^35^ leading to a collapse of the pristine crystal structure. This amorphisation was found to occur for strain approximately 16%. Literature values of the damage density, required to induce such a strain range between 1 × 10^22^ vacancies cm^−3^ ^36^ and 9 × 10^22^ vacancies cm^−3^ ^37^. The implantation fluences were low enough so that all of the damage densities explored in the present work are significantly smaller than these amorphisation thresholds, thus ruling out possible phase transitions in the sample, which is therefore to be considered as defective diamond for all implantation conditions.

**Figure 2:**
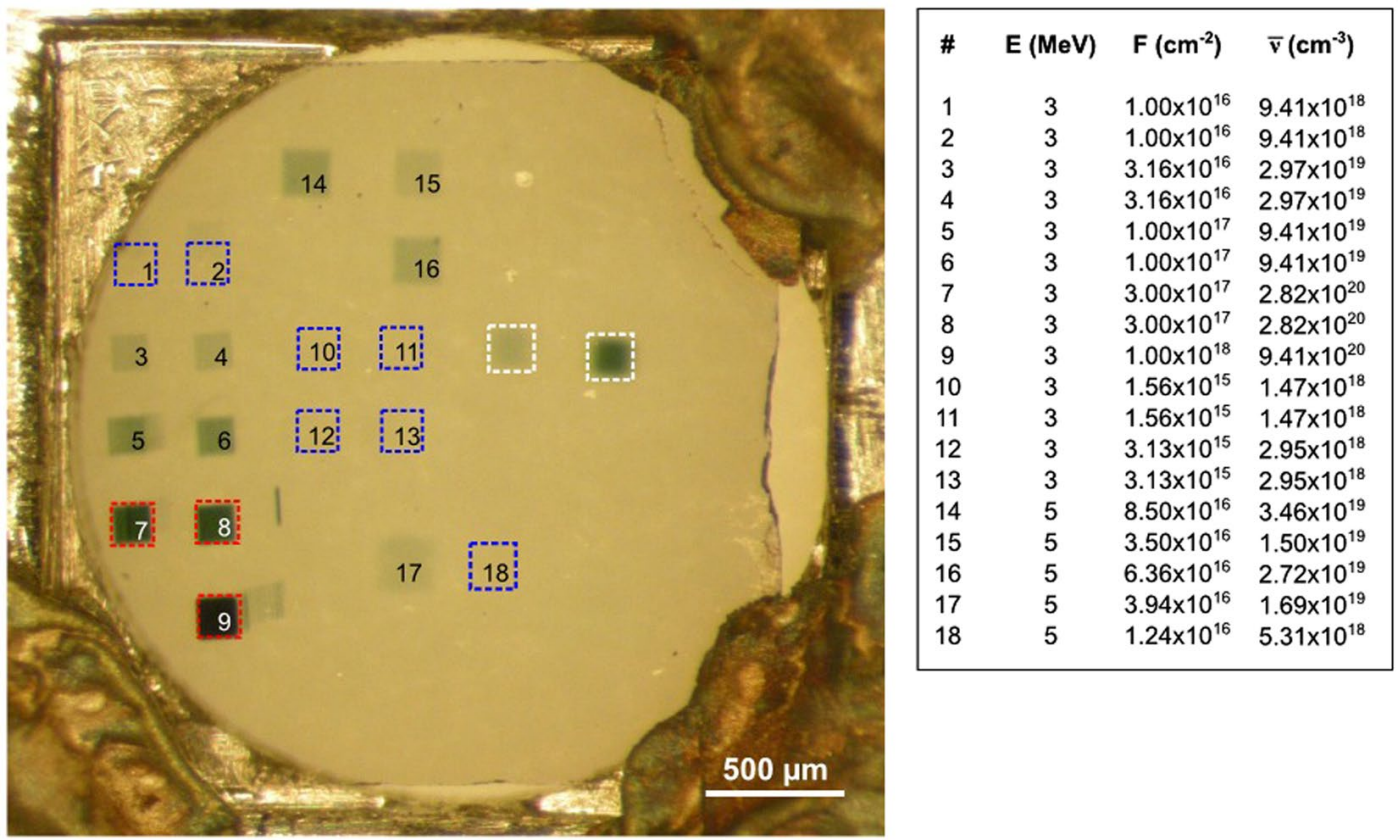
Optical transmission micrograph of the irradiated sample. The regions highlighted by red squares were irradiated at the highest fluence and are clearly distinguishable due to their opacity, while regions irradiated at lower fluences (some of which are highlighted by blue squares) are scarcely visible. Note that two test implantations (highlighted by white squares) were not employed in the subsequent data analysis due to a larger uncertainty on the fluence value. The ion energy *E*, fluence *F* and average vacancy density $\bar{\nu }$ corresponding to each region are reported in the table on the right.

Figure 2 shows an optical transmission micrograph of the sample after ion irradiation across the areas characterised by different fluences. Only the regions irradiated at higher fluences are clearly distinguishable due to their opacity, while the regions irradiated at lower fluences are not visible.

After proton irradiation, the sample was characterised with a laser interferometric microscope (Maxim 3D, Zygo Corporation) with the purpose of evaluating the variation of optical thickness of each irradiated area with respect to non-irradiated regions of the sample, (see the “Methods” Section for further details). The technique allows a nanometric resolution in the determination of the optical thickness variations, in ideal operating conditions.

Optical profilometry characterization (not reported here) demonstrated that the areas implanted at the highest fluences while still displaying an adequate transparency for the optical measurements (i.e.: 3 MeV H, fluence = 3 × 10^17^ cm^−2^) displayed a surface swelling^38^ of 2 nm. Such swelling value was barely measurable in the sample characterised by a ~2 nm surface roughness. Moreover, even under the reasonable assumption that the uniformly damaged thickness of the sample is bulging at both surfaces, a 4 nm swelling still accounts for a negligible fraction of the measured variation in Optical Path Difference (OPD). Therefore, damage-induced swelling effects can be neglected at these low damage densities. Consequently, we can assume that the phase shift observed in correspondence with the irradiated regions is caused by a variation of optical thickness which is entirely attributed to the variation of the refractive index of the material across its thickness.

**Figure 3:**
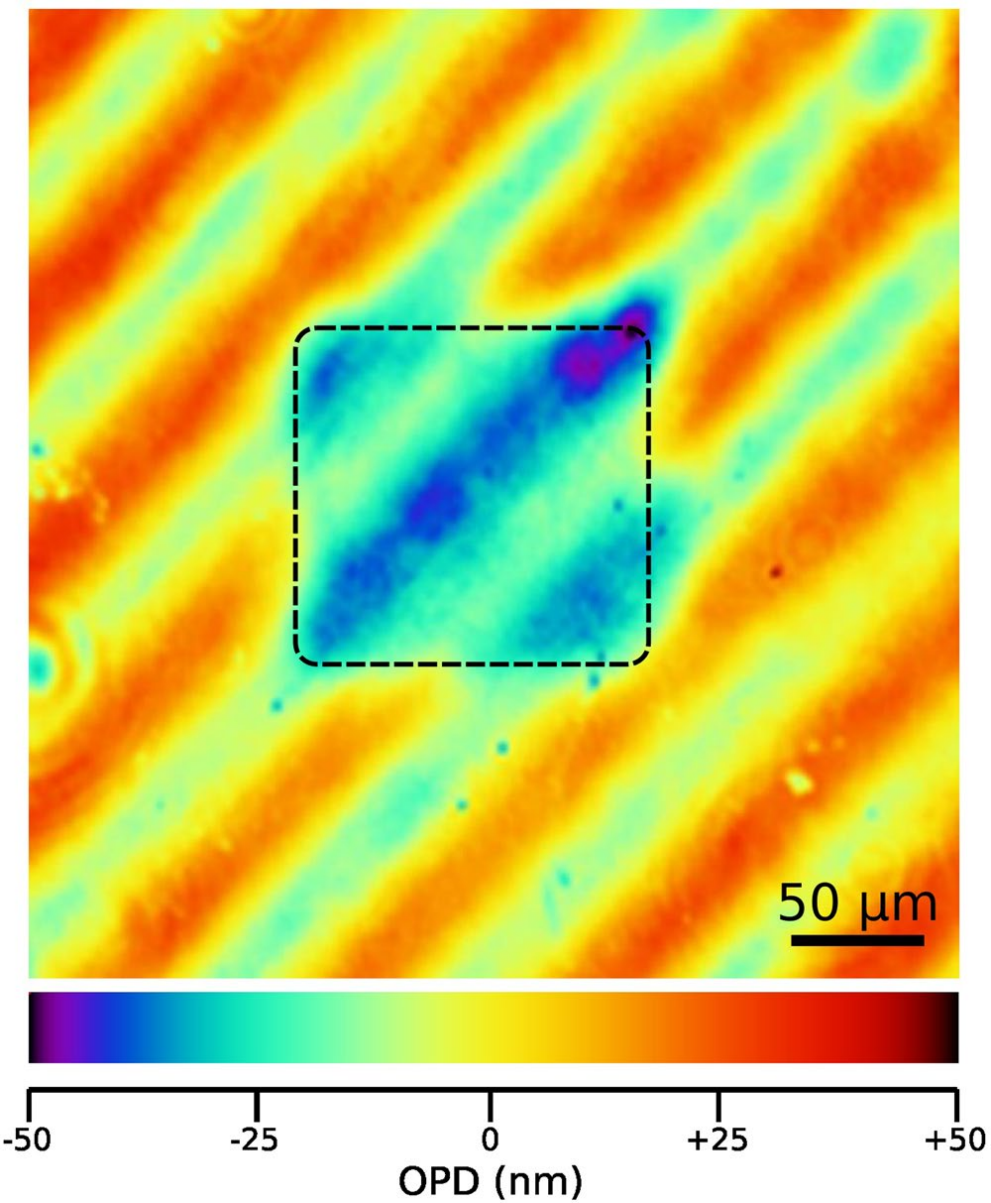
OPD micrograph. OPD map obtained by laser interferometric microscopy from a 125 × 125 μm^2^ region irradiated with 5 MeV H^+^ at a fluence of 8.7 × 10^16^ cm^−2^. The colour scale at the bottom encodes the measured OPD variation for each pixel. The irradiated region is highlighted by the dashed-line square and is characterized by a different optical thickness with respect to the surrounding region. Interference fringes due to multiple internal reflections are also clearly distinguishable in both the irradiated and non-irradiated areas.

In Fig. 3 a typical map of the OPD is reported for a 125 × 125 μm^2^ region irradiated with 5 MeV H^+^ at a fluence of 8.7 × 10^16^ cm^−2^. The edges of the irradiated region are highlighted by the dashed-line square and a clear OPD contrast between irradiated and non-irradiated regions can be appreciated. Also, interference fringes are clearly distinguishable in both the irradiated and non-irradiated areas. They are attributed to multiple internal reflections within the sample, and they are much more pronounced with respect to previous measurements with the same technique^22, 23^ due to the significantly smaller thickness of the sample. While determining the OPD variation for each irradiated region, care was taken to subtract this sinusoidal background from the experimental data. Also, for each irradiation, only the pixels well within the irradiated region (i.e. comprised in an area with ~75% lateral size with respect to the edges highlighted in Fig. 3) were considered, to avoid edge effects.

## Discussion

Consistently with previous works^22–24, 27–29^, SRIM-2008.04 Monte Carlo code^37^ was employed to numerically simulate the structural damage induced in the diamond sample by ion irradiation. The volumetric concentration of single vacancies *ν* was adopted as an effective parameter to quantify the induced damage density. This quantity was derived in a simple linear approximation as:

1 $$\nu (z)=F\cdot p(z)$$

where *z* is the depth coordinate across the sample thickness, *F* is the irradiation fluence and *p*(*z*) is the linear density of induced vacancies per incoming ion per unit length in the depth direction provided by the SRIM-2008.04 code^37^. In principle, the linear dependence of vacancy density on fluence does not take into account defect-defect interactions and consequently disregards the formation of multi-vacancy complexes. Nonetheless, for the low vacancy density values considered in this work, i.e. up to ~1‰ of the atomic density of the target crystal and far below the amorphization of diamond^35^, this approximation is perfectly adequate to describe a damage process which is dominated by the formation of isolated point defects^39^.

The numerical simulations were run by averaging over ensembles of 5 × 10^4^ ions and setting the atomic displacement energy to 50 eV^40, 41^. Unless otherwise stated, the reported simulations were consistently carried in “Detailed calculation with full damage cascade” mode as reported in Fig. 1. Occasionally, results obtained from simulations carried in “Quick calculation” mode will be provided, to compare them with the results reported in refs 22 and 23 for MeV proton implantations.

As reported in equation (1), the relevant profiles for the volumetric vacancy density for each irradiation can be derived by re-scaling the *p*(*z*) values of the linear vacancy concentration plotted in Fig. 1 by a factor corresponding to the irradiation fluence, while (as previously mentioned) no volumetric expansion effects were taken into account. Likewise, for each irradiation the average volumetric vacancy density $\bar{\nu }$ across the sample thickness can be obtained by rescaling $\bar{p}$ (i.e. $\bar{p}$ = 941 vac cm^−1^ per ion and $\bar{p}$ = 428 vac cm^−1^ per ion for 3 MeV and 5 MeV protons, respectively) by the corresponding implantation fluence.

As previously mentioned, no significant changes in the sample thickness are associated with the induced damage, therefore the induced structural damage determines a change in optical thickness which can be exclusively attributed to the variation of the refractive index. At low damage densities, this variation can be assumed to be linearly dependent upon from the vacancy density^22^. Consequently, we can express the OPD as:

2 $$OPD(\lambda )={\int }_{0}^{t}\Delta n(z,\lambda )dz=k(\lambda )\cdot F\cdot {\int }_{0}^{t}p(z)dz=k(\lambda )\cdot t\cdot F\cdot \bar{p}$$

where *t* is the sample thickness and *k* is the proportionality constant linking Δ*n* and *ν*.

Therefore, we obtain that the average refractive index variation within each irradiated region is directly proportional to the average volumetric vacancy density, i.e.:

3 $$\Delta \bar{n}(\bar{\nu },\lambda )=\frac{OPD(\lambda )}{t}=k(\lambda )\cdot \bar{\nu }$$

**Figure 4:**
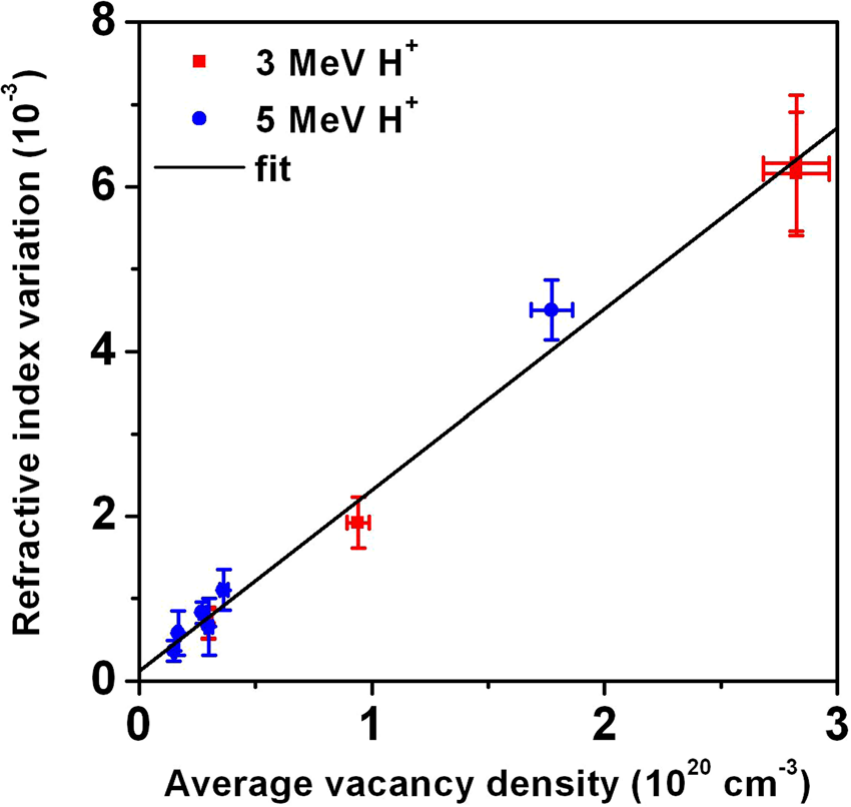
Refractive index variation. Plots of the average refractive index variation $\Delta \bar{n}$ measured at *λ* = 632.8 nm as a function of volumetric vacancy densities for both 3 MeV (red square dots) and 5 MeV (blue circular dots) proton irradiations. The black line reports the linear fitting of the whole dataset.

In Fig. 4 plots of the average refractive index variations *Δ* $\bar{n}$ measured at *λ* = 632.8 nm for different regions irradiated with both 3 MeV and 5 MeV protons are reported as functions of the corresponding average vacancy densities estimated from the relevant fluence and $\bar{p}$ values. As previously reported for MeV proton implantation^22, 23^, the refractive index variation exhibits a systematic linear increase as a function of induced damage. Regions irradiated at fluences below 3 × 10^16^ cm^−2^ could not be measured despite the high OPD sensitivity of the technique, due to the presence of background interference fringes. As far as the regions implanted at the highest damage densities are concerned, regions irradiated at fluences above 4 × 10^17^ cm^−2^ could not be measured due to their high opacity. It is worth noting that data relevant to the different ion energies are compatible within the experimental uncertainties, thus confirming the validity of the linear approximation that links irradiation fluence, vacancy density and refractive index variations. Therefore, by performing a single linear fitting procedure on all reported data, it is possible to estimate the proportionality factor *k* linking the induced structural damage (parameterised by the volumetric vacancy density) and the refractive index variation at *λ* = 632.8 nm as *k* = (2.20 ± 0.14) × 10^−23^ cm^3^.

For the sake of consistency with previous reports on MeV proton implantation^22, 23^, the vacancy density values were also estimated in “Quick damage calculation” mode, yielding through the same analysis procedure an estimation of the proportionality factor *k* = (3.0 ± 0.2) × 10^−23^ cm^3^. This value shows a statistically significant difference with respect to the previously reported value of *k* = (4.34 ± 0.05) × 10^−23^ cm^3^ ^23^, in which most of the irradiated ions were implanted into the sample bulk. This is attributed to the effects of (i) strong refractive index gradients occurring at the end-of-range damage peak, (ii) doping effects from implanted ions and/or (iii) complex effects due to the interplay between electronic and nuclear energy loss occurring at the ion end of range^33^. For the above-mentioned reasons, we regard the current estimation of the Δ*n*/*ν* proportionality factor as more accurate and general, since in this work a significantly more uniform refractive index variation profile was induced through the sample thickness while substantially minimising the implantation of the irradiated ions within the target material.

Generally, the refractive index variation in a structurally modified material can be effectively described by the Wei adaptation of the Lorentz-Lorenz equation:

4 $$\frac{\Delta {\rm{n}}}{n}=\frac{({n}^{2}-1)({n}^{2}+2)}{6{n}^{2}}\cdot [-\frac{\Delta V}{V}+\frac{{\rm{\Delta }}\alpha }{\alpha }+f]$$

where *V* is volume, *α* is polarizability and *f* is the structure factor of the target implanted material^42^. Structural damage in a crystalline material typically results in a volumetric expansion due to the lower atomic density of the partially amorphised material with respect to the pristine crystalline phase, and therefore in a reduction of the refractive index as expressed in equation (4). In most of the cases, the volumetric expansion is the dominant effect determining the refractive index variation, but for specific crystals the breaking of chemical bonds can result in an increased polarizability, which in turn determines an increasing refractive index value. In the present study, as mentioned above, a negligible volumetric expansion was measured as compared to variations in optical thickness. Instead, structural damage in diamond results in the breaking of strongly covalent *sp* ^*3*^ chemical bonds in favour of the formation of *sp* ^*2*^ chemical bonds. Therefore, it is reasonable to attribute the refractive index variation to the predominant effect of changes in the atomic polarizability, as confirmed by the increasing trend of such a variation. It is worth noting that previous results exhibiting a non-monotonic variation of the refractive index as a function of implantation fluence^28, 29^ were obtained upon the implantation of heavier (Ga) ions which were implanted into the target material and caused non-negligible swelling effects. For these reasons, the simple model employed in this work would not be suitable to describe the non-monotonic trends reported in refs 28 and 29, since the effect of concurrent volumetric expansion (and possibly the effects of the implanted atoms themselves) needs to be suitably taken into account.

To summarise, the ion-damage-induced refractive index variation in single crystal diamond was systematically investigated with laser interferometric microscopy in a 38 µm thick CVD sample implanted with 3 MeV and 5 MeV protons at increasing fluences in the 3 × 10^16^–4 × 10^17^ cm^−2^ range. Consistently with previous reports on MeV proton implantation^22, 23^, the refractive index in the irradiated area exhibited a systematic linear increase as a function of induced damage density, with consistent trends for different proton energies. The reduced thickness of the sample combined with the high penetration depth of the employed ion beams allowed the deposition of extremely uniform damage depth profiles, while minimising the actual implantation of the accelerated ions in the target material. This resulted in a more accurate estimation of the proportionality factor linking refractive index variation and the damage density induced by MeV proton irradiation. Moreover, swelling effects in the irradiated material could be ruled out, thus allowing the unequivocal attribution of the observed refractive index variation to changes in the polarizability of the material. This contributed to shed light in the interpretation of previous contradictory results.

These results can provide a useful guide in the fabrication of buried waveguides and the fine tuning of optical cavities, provided that the possible side-effects of the implantation process other than the polarizability variations (i.e.: volumetric variations, doping effects) are suitably taken into account.

## Methods

**Figure 5:**
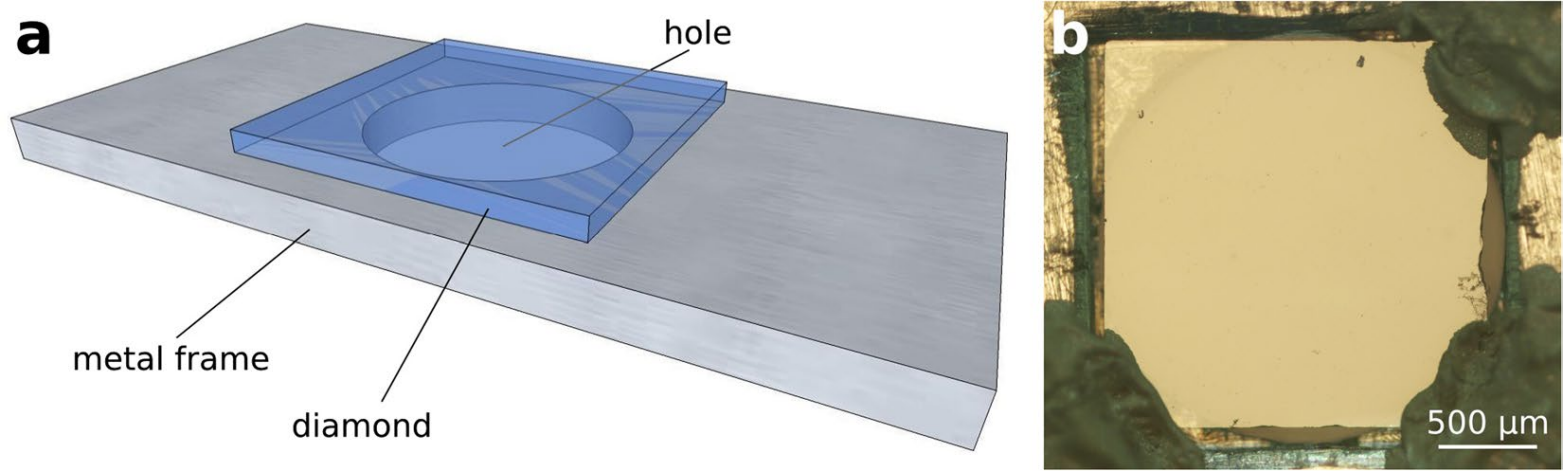
Sample schematics. (**a**) Schematic representation of the mounting of the thin diamond sample on a metallic frame; the drawing is not to scale. (**b**) Optical micrograph of the mounted sample in frontal geometry.

The sample under investigation is a mechanically thinned type-IIa single crystal CVD diamond produced by ElementSix. The crystal orientation of its frontal surface was (100). The thickness of the sample was estimated by optical means as (38 ± 2) µm, while its surface roughness was measured by white-light optical interferometry microscopy as ~2 nm rms. The sample was fixed with silver paint on a metallic frame for ease of handling, as shown in Fig. 5. From optical microscopy in cross-polarised beams, no evidence of birefringence was found, so that it was possible to rule out significant stress fields in the sample due to thinning, polishing and mounting.

The sample was irradiated at room temperature with proton microbeams at the MP2 microbeam line of the NEC 5U Pelletron accelerator of the MARC Centre in Melbourne (3 MeV implantations)^43^ and the external microbeam line of the 3 MV Tandetron accelerator of the INFN LABEC Laboratory in Florence (5 MeV implantations)^44, 45^. In both cases, irradiations at different fluences were carried out by raster-scanning proton beams with sizes between 1 μm and 10 μm over ~125 × 125 μm^2^ areas to deliver a homogeneous fluence over the central area of each irradiated region^23^. While the former implantation run was conducted under high-vacuum conditions, the latter one was performed by extracting the ion beam in standard atmosphere. In both cases, the sample was kept at an angle of ~5° with respect to the direction of the incident beam, to avoid channeling effects. Typical ion currents were in the 0.1–1.0 nA and 0.5–1.5 nA ranges for 3 MeV H^+^ and 5 MeV H^+^ irradiations, respectively. For 3 MeV H^+^ irradiations, the fluence was estimated by directly measuring the ion current with a Faraday cup prior to each irradiation and consequently timing each irradiation time; care was taken to measure the ion current after each irradiation, to check the beam current stability. For 5 MeV H^+^ irradiation, fluence calibration was performed by preliminarily correlating the ion current measured with a Faraday cup with the yield of ion-beam-induced characteristic X-rays from the exit window of the external beam, and subsequently by monitoring the X-ray yield during irradiation (see refs 23 and 46 for further details). After ion implantation, the sample was not subjected to any post-processing procedure, due the extreme fragility of both the diamond layer and its mounting on the supporting metallic frame.

**Figure 6:**
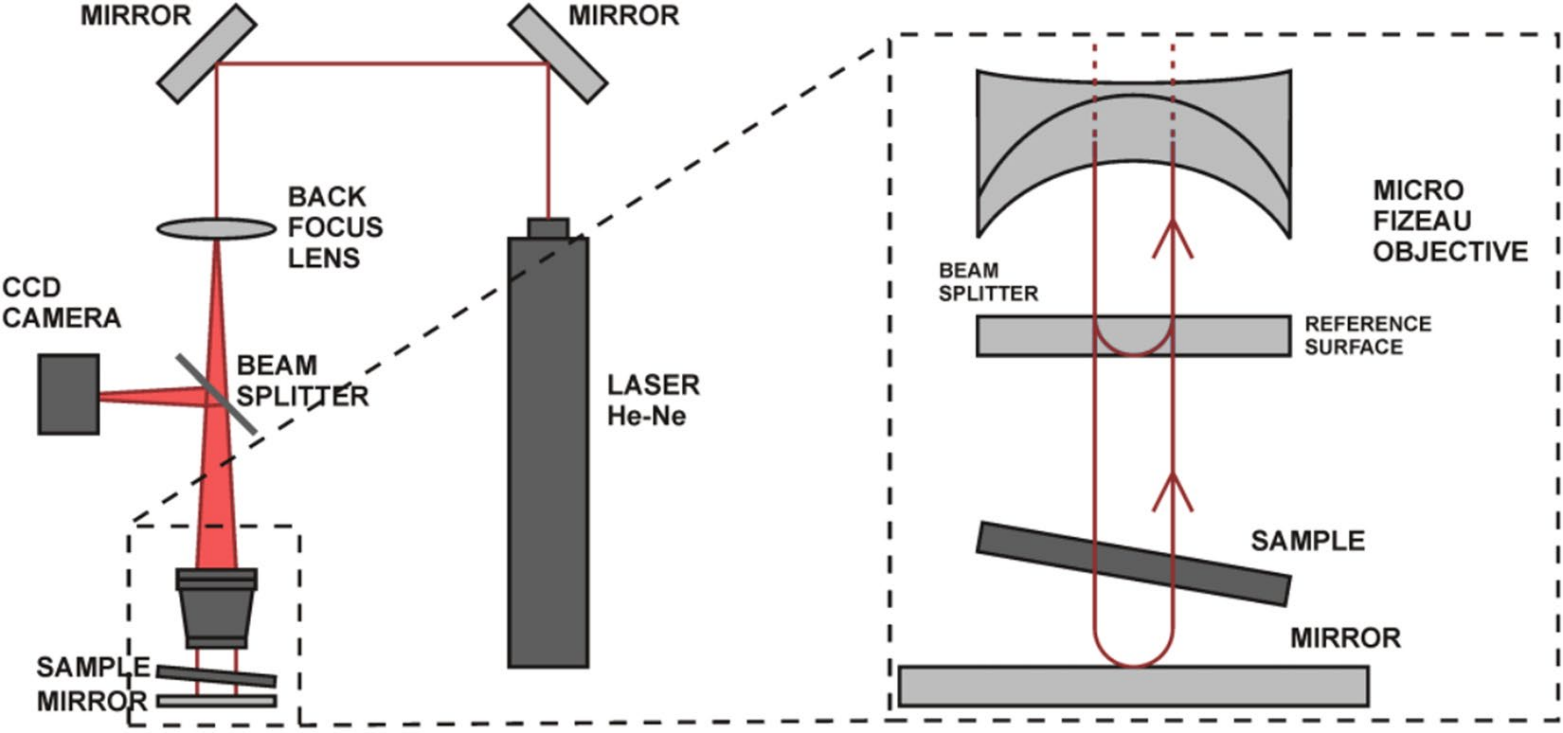
Laser interferometric microscope. Schematic representation of the operating principle of the laser interferometric microscope.

As schematically shown in Fig. 6 and reported in further experimental details in ref. 23, a *λ* = 632.8 nm probe beam from a He-Ne laser was employed in conjunction with a 20× micro-Fizeau objective. By means of the phase-shift method^47^, the relative phase of the test beam crossing the sample with respect to the reference beam was reconstructed at each pixel with a lateral spatial resolution better than 2 µm and with a field view of 349 × 317 μm^2^. It is worth noting that the sample was mounted in a tilted position to reduce the effects of undesired internal reflections between the two opposite surfaces of the sample.
